# Rapid Emergence and Clonal Dissemination of CTX-M-15–Producing *Salmonella enterica* Serotype Virchow, South Korea

**DOI:** 10.3201/eid2201.151220

**Published:** 2016-01

**Authors:** Jin Seok Kim, Young-Sun Yun, Soo Jin Kim, Se-Eun Jeon, Deog-yong Lee, Gyung Tae Chung, Cheon-Kwon Yoo, Junyoung Kim, PulseNet Korea Working Group

**Affiliations:** Korea National Institute of Health, Cheongju-si, South Korea

**Keywords:** extended-spectrum β-lactamases, ESBL, Salmonella enterica, CTX-M-15, Salmonella enterica serotype Virchow, IncHI2, emergence, clonal dissemination, S. enterica, South Korea, bacteria, prevalence, nontyphoidal salmonella, antimicrobial resistance, surveillance, salmonella

## Abstract

The prevalence of cefotaxime-resistant *Salmonella enterica* serotype Virchow has dramatically increased in South Korea since the first isolation in 2011. Of 68 isolates collected over 10 years, 28 cefotaxime-resistant isolates harbored the *bla*_CTX-M-15_ extended-spectrum β-lactamase gene and were closely related genetically, demonstrating the clonal dissemination of CTX-M-15–producing *Salmonella* Virchow in South Korea.

Nontyphoidal salmonella, a foodborne pathogen, causes human gastroenteritis worldwide. Among >2,500 different *Salmonella enterica* serotypes, *Salmonella* Enteritidis and *Salmonella* Typhimurium are the most common serotypes responsible for human salmonellosis (*1*). In Europe, *Salmonella* Virchow has recently increased in prevalence, and a high proportion of isolated strains are resistant to multiple antimicrobial drugs (*2*–*4*).

Third-generation cephalosporins are widely used to treat major bacterial infections in humans and animals (*5*). However, the emergence and rapid spread of drug-resistant bacteria has become a serious public health concern. Extended-spectrum β-lactamases (ESBLs) are known to confer antimicrobial drug resistance by hydrolyzing most β-lactam antimicrobial drugs, including third-generation cephalosporins (*5*). Since the first report from Spain in 2000 of strains producing CTX-M-9 (*6*), which confers resistance to cefotaxime, various CTX-M–type ESBLs have been identified in *Salmonella* Virchow. In Spain, Belgium, and France, CTX-M-9 and CTX-M-2 producers spread clonally in humans and poultry (*7*,*8*). In addition, the *bla*_CTX-M-15_ gene was identified in porcine isolates in South Korea (*9*). These reports demonstrate that CTX-M–producing *Salmonella* Virchow clones can be easily transmitted to humans through food products of animal origin. In South Korea, the incidence of *Salmonella* Virchow infections in humans has increased over the years, necessitating a nationwide survey of antimicrobial drug resistance in *Salmonella* Virchow isolates.

## The Study

During 2005–2014 in South Korea, local public health laboratories participating in the national surveillance network isolated 68 *Salmonella* Virchow strains from feces samples from patients with acute diarrhea. Until 2010, <5 *Salmonella* Virchow strains were isolated per year, but this number gradually increased to 17 in 2014 (Figure). *Salmonella* Virchow consistently ranked among the top 10 serotypes in prevalence during each study year in South Korea, accounting for ≈1.5%–2% of salmonellosis cases.

**Figure:**
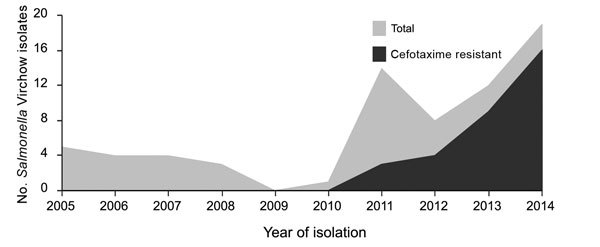
Temporal distribution of *Salmonella enterica* serotype Virchow isolates in South Korea, 2005–2014.

We used the broth microdilution method (*10*) to perform antimicrobial susceptibility testing of *Salmonella* Virchow; results showed that 54 (79.4%) of the 68 isolates were resistant to >1 of the 15 antimicrobial agents tested (Table; Technical Appendix Figure). The highest resistance rates were noted for nalidixic acid (77.9%), followed by ampicillin (44.1%), cefotaxime (44.1%), tetracycline (42.6%), and gentamicin (23.5%). Only 2 (2.9%) isolates were resistant to ciprofloxacin, but 50 (73.5%) had intermediate resistance. All of the isolates were susceptible to chloramphenicol, amikacin, or imipenem. Multidrug resistance, defined as resistance to at least 3 different classes of antimicrobial agents, was found in 30 (44.1%) isolates (Table).

**Table:** Antimicrobial resistance profiles of *Salmonella enterica* serotype Virchow isolates in South Korea, 2005–2014*

Isolate no.	Antimicrobial drug	No. resistant isolates
1	–	14
2	NAL	18
3	TCY	2
4	NAL, SXT	2
5	TCY, NAL	2
6	AMP, CEF, CTX, NAL	5
7	AMP, CEF, CTX, NAL, TCY	7
8	AMP, CEF, CTX, GEN, NAL	1
9	AMP, CEF, CTX, GEN, NAL, TCY	15
10	AMC, AMP, CEF, CTX, FOX, SAM, NAL	1
11	AMC, AMP, CEF, CTX, FOX, SAM, NAL, TCY	1
Total		68

*AMC, amoxicillin/clavulanic acid; AMP, ampicillin; CEF, cephalothin; CTX, cefotaxime; FOX, cefoxitin; GEN, gentamicin; NAL, nalidixic acid; SAM, ampicillin/sulbactam; SXT, trimethoprim/sulfamethoxazole; TCY, tetracycline; –, pansusceptible.

All multidrug-resistant isolates showed resistance to third-generation cephalosporins. In South Korea, cefotaxime-resistant strains were first isolated in 2011. Since then, 4, 9, and 14 isolates were collected in 2012, 2013, and 2014, respectively (Figure). The rates of cefotaxime resistance in *Salmonella* Virchow have increased markedly, from 21.4% in 2011 to 82.3% in 2014. This annual trend of increasing cefotaxime resistance in South Korea is of interest because the rates were substantially higher than those reported in Spain during 2002–2006 (15%) (*11*) and Belgium during 2009–2013 (<10%) (*12*). Moreover, even in Israel and Switzerland, where the incidence of *Salmonella* Virchow was relatively higher than that in South Korea, antimicrobial drug resistance to third-generation cephalosporins was rare (*3*,*4*).

Among the 30 cefotaxime-resistant *Salmonella* Virchow isolates, 28 were confirmed to be ESBL-producers. PCR and sequencing of β-lactamase genes (*13*) confirmed that these 28 isolates harbored a *bla*_CTX-M-15_ gene, whereas the other 2 contained a *bla*_CMY-2_ gene. Cefotaxime resistance was transferred by conjugation from 9 *Salmonella* Virchow isolates to *Escherichia coli* J53 recipients, and the *bla*_CTX-M-15_ gene was identified in transconjugants. Southern blotting and PCR-based replicon typing (*14*) showed that all plasmids in transconjugants were ≈215 kb in size and possessed an IncHI2 plasmid, which was further assigned to sequence type (ST) 2 by plasmid double locus sequence typing (*15*). The analysis of the genetic environment surrounding the *bla*_CTX-M-15_ gene (*13*) in transconjugants showed that insertion sequences IS*Ecp1* and o*rf477* were detected 48 bp upstream and downstream of the *bla*_CTX-M-15_ gene, respectively. This IS*Ecp1*-*bla*_CTX-M-15_–open reading frame 477 transposable unit was also identified in other incompatibility groups of the plasmids in *Enterobacteriaceae*. Furthermore, it was identical to that of the ST2-IncHI2 plasmid of *Salmonella* Enteritidis isolated from humans and poultry meat in South Korea (J. Kim, unpub. data), suggesting that the *bla*_CTX-M-15_ gene in *Salmonella* Virchow might have originated from IS*Ecp1*-mediated transposition followed by interspecies spread through the IncHI2-type plasmid studied here.

All of the CTX-M-15–producing strains had reduced ciprofloxacin susceptibility (MICs of 0.25–0.5 µg/mL). All 10 randomly selected isolates harbored a single substitution within the quinolone resistance–determining region of GyrA at codon 83 (Ser→Phe), which is the major mutation described in *Salmonella* species (*8*). Because fluoroquinolones and third-generation cephalosporins are the drugs of choice for treating severe *salmonella* infections in humans, the reduced ciprofloxacin susceptibility in cefotaxime-resistant *Salmonella* Virchow is considered a critical risk factor for infections with these strains.

The genetic relationship of the 68 *Salmonella* Virchow isolates was determined by using multilocus sequence typing (http://mlst.warwick.ac.uk/mlst/dbs/Senterica) and pulsed-field gel electrophoresis (PFGE) according to a standardized protocol. Seven multilocus sequence typing loci displayed 4 different profiles, and most isolates belonged to sequence type (ST) 16 (n = 59), followed by ST197 (n = 6), ST359 (n = 2), and ST426 (n = 1). All of the 28 CTX-M-15–producing strains were typed as ST16, but the cefotaxime-susceptible isolates were also assigned to this type. The PFGE analysis yielded sufficient discriminatory power in typing *Salmonella* Virchow isolates; 22 *Xba*I and 21 *Bln*I PFGE patterns were generated. Although the isolates shared >70% similarity, CTX-M-15–producing strains clustered on the basis of a similarity value of 90% (Technical Appendix Figure), indicating the clonality of cefotaxime-resistant strains.

For humans, the main route of *Salmonella* infection is the consumption of contaminated food of animal origin, and *Salmonella* Virchow is one of the most prevalent serotypes identified in poultry and poultry products. The use of cephalosporins in animal production has led to emergence of antimicrobial drug–resistant *Salmonella* Virchow clones among food animals (*8*), posing a threat to public health because of the possible transmission of these bacteria through the food chain. In fact, the *Xba*I PFGE pattern identified among human isolates in this study was identical to that in 2 cefotaxime-resistant *Salmonella* Virchow strains isolated in 2012 from poultry meat in our collection (Technical Appendix Figure). Furthermore, this pattern appeared similar to the patterns of *Salmonella* Virchow harboring the *bla*_CTX-M-15_ gene on an ST2-IncHI2 plasmid that was isolated from organically raised pigs in South Korea (*9*). Resistant clinical strains were collected from 13 of 15 local public health laboratories in South Korea during 2011–2014; thus, although the mode of the spread of *Salmonella* Virchow in humans is not established, it has been postulated that CTX-M-15–producing *Salmonella* Virchow might have disseminated clonally through the nationwide distribution of contaminated food products rather than through independent emergence.

## Conclusions

We analyzed the antimicrobial drug susceptibility and genetic relatedness of *Salmonella* Virchow isolates from patients with diarrhea in South Korea. Of 68 isolates obtained during 2005–2014, a total of 30 were resistant to third-generation cephalosporins. The prevalence rate of the resistant strains has dramatically increased since the isolation of cefotaxime-resistant strains in 2011. These findings suggest that the cefotaxime-resistant isolates are genetically closely related and harbor a plasmid carrying the *bla*_CTX-M-15_ gene of the same compatibility group (ST2-IncHI2), representing clonal dissemination of CTX-M-15–producing *Salmonella* Virchow in South Korea and posing an urgent threat to public health. Therefore, more comprehensive surveillance is required to prevent further spread of resistant clonal strains.

Technical Appendix Composite dendrogram of the genetic relatedness among *Salmonella enterica* serotype Virchow isolates in Republic of Korea.
