# Supplementary material for: Rapid Emergence and Clonal Dissemination of CTX-M-15–Producing Salmonella enterica Serotype Virchow, South Korea
Source: Emerg Infect Dis. 2016 Jan;22(1):68–70. doi: 10.3201/eid2201.151220 (PMC4696704; doi:10.3201/eid2201.151220)
Supplement: Technical Appendix — Composite dendrogram of the genetic relatedness among Salmonella enterica serotype Virchow isolates in Republic of Korea. [file 15-1220-Techapp-s1.pdf]

Article DOI: http://dx.doi.org/10.3201/eid2201.151220

Rapid Emergence and Clonal Dissemination of CTX-M-15–Producing *Salmonella enterica* Serotype Virchow, South Korea

Technical Appendix

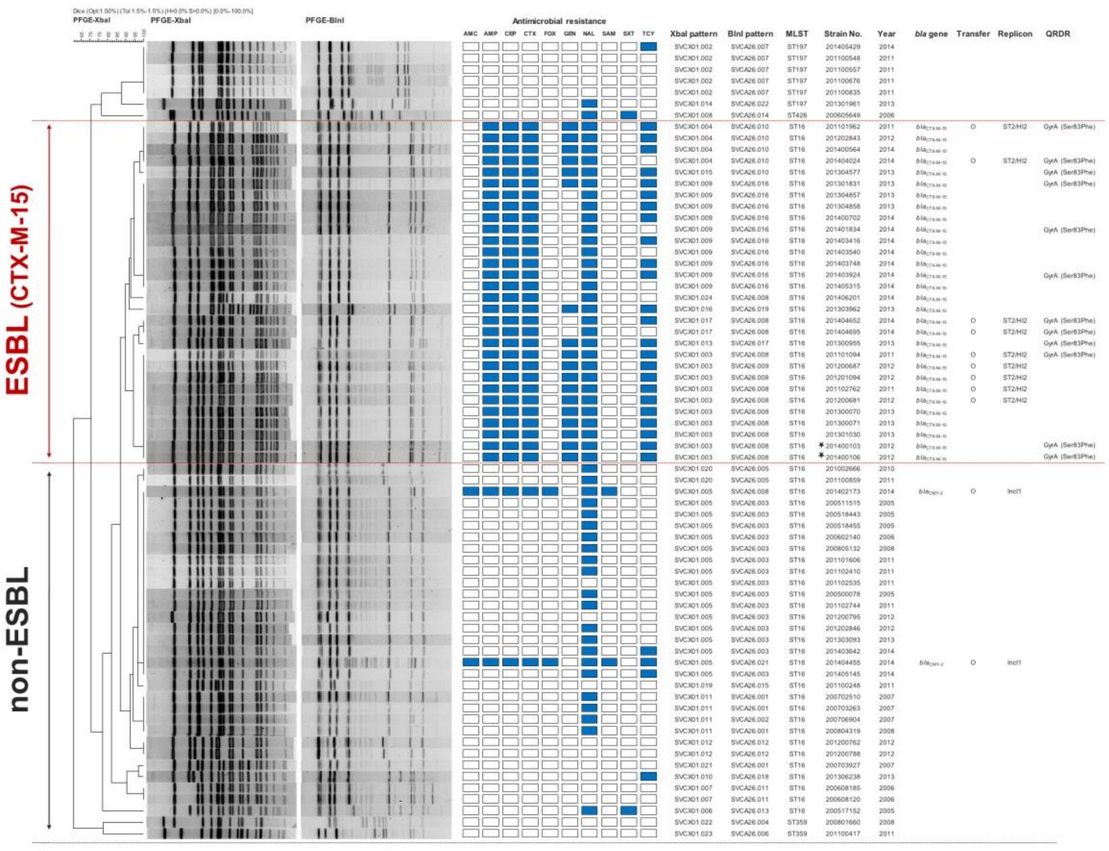

Technical Appendix Figure. Composite dendrogram of the genetic relatedness among *Salmonella enterica* serotype Virchow isolates in South Korea. Clustering was calculated using the BioNumerics v5.1 software (Applied Maths, Austin, TX, USA), based on the Dice coefficient and UPGMA with a 1.5% band position tolerance and 1.5% optimization. The *Xba*I and *Bln*I PFGE pattern names were assigned by the Korea PulseNet database. The antimicrobial resistance, MLST, characteristics of the plasmids harboring

the *bla* genes and quinolone resistance determinant are shown next to the PFGE profiles. Two additional chicken isolates (indicated with asterisks) are included for comparison. AMC, amoxicillin/clavulanic acid; AMP, ampicillin; CEP, cephalothin; CTX, cefotaxime; FOX, cefoxitin; GEN, gentamicin; NAL, nalidixic acid; SAM, ampicillin/sulbactam; SXT, trimethoprim/sulfamethoxazole; TCY, tetracycline; QRDR, quinolone resistance-determining region.
